# A Homogeneous, High-Throughput Assay for Phosphatidylinositol 5-Phosphate 4-Kinase with a Novel, Rapid Substrate Preparation

**DOI:** 10.1371/journal.pone.0054127

**Published:** 2013-01-10

**Authors:** Mindy I. Davis, Atsuo T. Sasaki, Min Shen, Brooke M. Emerling, Natasha Thorne, Sam Michael, Rajan Pragani, Matthew Boxer, Kazutaka Sumita, Koh Takeuchi, Douglas S. Auld, Zhuyin Li, Lewis C. Cantley, Anton Simeonov

**Affiliations:** 1 National Institutes of Health Chemical Genomics Center, National Center for Advancing Translational Sciences, National Institutes of Health, Rockville, Maryland, United States of America; 2 Beth Israel Deaconess Medical Center, Department of Medicine, Division of Signal Transduction; Department of Systems Biology, Harvard Medical School, Boston, Massachusetts, United States of America; 3 Division of Hematology and Oncology, Department of Internal Medicine, Neuroscience Institute: Brain Tumor Center, University of Cincinnati, College of Medicine, Cincinnati, Ohio, United States of America; 4 Biomedicinal Information Research Center, National Institute of Advanced Industrial Science and Technology, Koto, Tokyo, Japan; 5 Center for Proteomic Chemistry, Novartis Institutes for Biomedical Research, Cambridge, Massachusetts, United States of America; University of Edinburgh, United Kingdom

## Abstract

Phosphoinositide kinases regulate diverse cellular functions and are important targets for therapeutic development for diseases, such as diabetes and cancer. Preparation of the lipid substrate is crucial for the development of a robust and miniaturizable lipid kinase assay. Enzymatic assays for phosphoinositide kinases often use lipid substrates prepared from lyophilized lipid preparations by sonication, which result in variability in the liposome size from preparation to preparation. Herein, we report a homogeneous 1536-well luciferase-coupled bioluminescence assay for PI5P4Kα. The substrate preparation is novel and allows the rapid production of a DMSO-containing substrate solution without the need for lengthy liposome preparation protocols, thus enabling the scale-up of this traditionally difficult type of assay. The Z’-factor value was greater than 0.7 for the PI5P4Kα assay, indicating its suitability for high-throughput screening applications. Tyrphostin AG-82 had been identified as an inhibitor of PI5P4Kα by assessing the degree of phospho transfer of γ-^32^P-ATP to PI5P; its inhibitory activity against PI5P4Kα was confirmed in the present miniaturized assay. From a pilot screen of a library of bioactive compounds, another tyrphostin, I-OMe tyrphostin AG-538 (I-OMe-AG-538), was identified as an ATP-competitive inhibitor of PI5P4Kα with an IC_50_ of 1 µM, affirming the suitability of the assay for inhibitor discovery campaigns. This homogeneous assay may apply to other lipid kinases and should help in the identification of leads for this class of enzymes by enabling high-throughput screening efforts.

## Introduction

**Figure 1:**
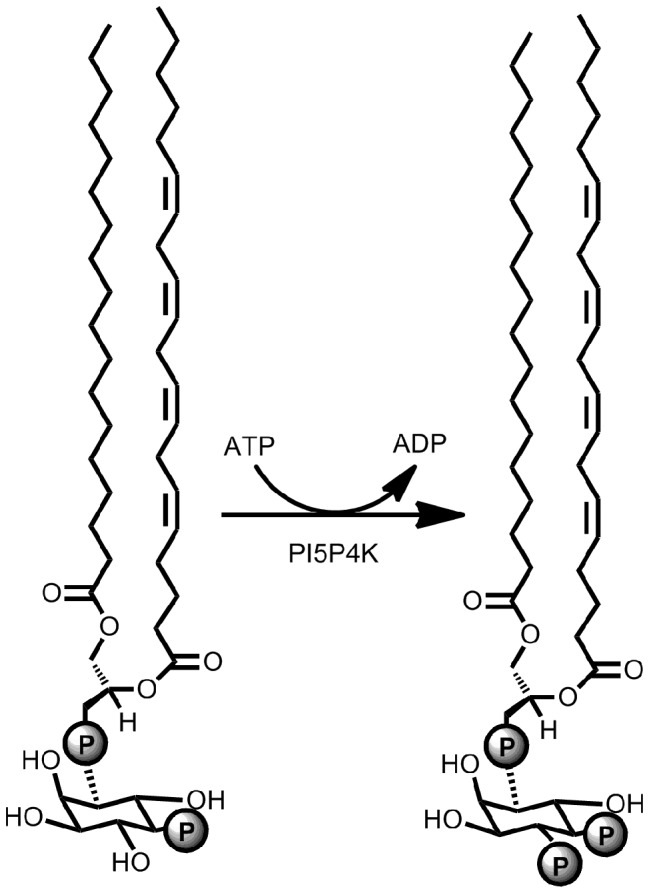
Schematic representation of the PI5P4K reaction using PI5P as the substrate. The additional carrier substrate DPPS is not shown.

Phosphatidylinositol (PI) signaling has been shown to impact a variety of fundamental cellular processes, including intracellular membrane trafficking, cytoskeletal rearrangement, cell proliferation, survival and growth. Dysregulation of these pathways can lead to cancer and other diseases [1], [2], [3], [4], [5], [6]. Phosphoinositides contain two fatty-acid chains linked through a diacylglycerol moiety and phosphodiester bond to an inositol headgroup. PIs are an important class of lipids that are regulated by reversible phosphorylation of the inositol headgroup. Phosphatidylinositides have three main phosphorylation sites on the inositol (positions 3, 4 and 5) that are regulated by different classes of phosphoinositide kinases and phosphatases. The three phosphatidylinositol mono-phosphates (PIPs) are PI3P, PI4P and PI5P. Importantly, these regioisomers have distinct roles *in vivo*, and there are three types of kinases (PIPKs) that distinguish and phosphorylate specific PIPs [7], [8], [9]. Type I PIP kinases (PI4P 5-kinases/PI4P5Ks) preferentially phosphorylate PI4P on the 5 position, Type III PIP kinases (PI5P 3-kinases/PI3P5Ks/Fab1/PIKfyve) preferentially phosphorylate PI3P on the 5 position, and Type II kinases (PI5P 4-kinases/PI5P4K), which are the focus of the present report, preferentially phosphorylate PI5P on the 4 position as shown in Figure 1 [10].

There has been a massive expansion in the investigation of the function and role of PI3K after the discovery of the phosphoinositide-3-kinase (PI3K) inhibitors wortmannin and LY294002 [11], [12]. While PI5P4K is known to play a role in insulin signaling, megakaryocyte development, and Vitamin-D signaling [13], [14], [15], [16], to date there are no commercially-available PI5P4K inhibitors, which hinders the advancement of this field. Recently, Demian *et al.* have demonstrated that selective inhibitors against PI5P4K can be obtained from screening [17]. However, potent PI5P4K inhibitors are still not available and such compounds would serve as valuable research tools to investigate the physiological role of PI5P4K activity.

The PIPKs share very little sequence homology with protein or other lipid kinases, which may facilitate the design of selective inhibitors. PI5P4Kβ has been crystallographically characterized and shows a flattened lysine-containing basic patch that is expected to bind to the phospholipid headgroup on the surface of the lipid membrane [18], [19]. PI5P4Kα and PI5P4Kγ also have crystal structures available (PDB ID:2YBX and 2GK9), and they show a similar overall structure. A binding assay and an enzymatic assay that utilized an elaborate liposome-based substrate preparation have been previously reported [17], [18]. Additionally, low-throughput radiolabeled enzymatic thin layer chromatography (TLC) assays were developed where the substrate was prepared in an isotonic KCl solution or as a liposome mixture [20], [21]. The radiometric assay uses γ-^32^P-ATP and PI5P and measures radiolabeled enzymatic product, PI(4,5)P_2_ after the separation by TLC [10], [22], [23]. There is, however, a need for additional assay types with simplified reproducible substrate preparations that are amenable to high-throughput screening.

We sought to establish a 1536-well compatible high-throughput enzymatic assay for PI5P4Kα to enable large multi-day library screens. A novel substrate preparation was developed that yielded a stable solution amenable to large-scale screening and that could be prepared reproducibly in house with common laboratory tools. Furthermore, the new assay was employed in a small library screen, resulting in the identification of an ATP-competitive tyrphostin (TYRosine PHOSphorylation INhibitors) inhibitor of PI5P4Kα (IC_50_ = 1 µM). This new high-throughput screening methodology should enable large library screening to help identify selective inhibitors of PI5P4Kα and related enzymes.

## Materials and Methods

### Reagents and Consumables

Hepes, pH 7.4 (4-(2-Hydroxyethyl)-1-piperazine ethanesulfonic acid) and MgCl_2_ were purchased from GIBCO (Carlsbad, CA, USA) and Quality Biological Inc. (Gaithersburg, MD, USA), respectively. EGTA, tyrphostin I-OMe-AG-538 and CHAPS (3-[(3-Cholamidopropyl)dimethylammonio]propanesulfonic acid) were purchased from Sigma-Aldrich (St. Louis, MO, USA). Dimethyl sulfoxide (DMSO, certified ACS grade) was obtained from Thermo-Fisher Scientific (Pittsburg, PA, USA). Tyrphostin 25 (AG-82) was purchased from Cayman Chemical Company (Ann Arbor, MI, USA). Medium binding white solid-bottom 1536-well plates (assay plates), 1536-well polypropylene plates (compound plates) and 384-well white solid-bottom plates were purchased from Greiner Bio One (Monroe, NC, USA). The 384-well polypropylene V-bottom plates (compound storage) were from Matrix/Thermo Scientific (Hudson, NH, USA). Bioluminescent assay detection used the Promega ADP-Glo kit (Madison, WI, USA), which came with 10 mM Ultrapure ATP that was used for the assay. Per the manufacturer’s protocol, this kit can be used for reactions containing between 1 µM to 1 mM ATP. PI5P4Kα with a GST tag was expressed and purified as described previously [10]. A 0.4 mg/mL stock was stored at −80°C and used for this work.

### Compound Library

The library of pharmacologically active compounds (LOPAC^1280^, Sigma-Aldrich) contains 1280 known bioactives that were received as 10 mM DMSO solutions. The compound library was plated in 1536-well format with an Evolution P^3^ (EP^3^) liquid dispenser (Perkin Elmer, Shelton, CT, USA). The library was formatted into columns 5–48 of 1536-well compound plates at 4 stock concentrations (1∶ 5 dilution, spanning the 10000 to 80 µM range) and 5 µL per well. Preparation of the compound library for quantitative high-throughput screening (qHTS) has been described previously [24]. A control plate was made in a compound plate in columns 1–4 using a Cybi-Well (CyBio, Jena, Germany) to transfer solutions from a 384 compound storage plate to the 1536 plate compound plate. Columns 1, 3, and 4 contained DMSO and column 2 contained a 1∶2 serial dilution of the control compound tyrphostin AG-82 (16 points with N = 2 and starting concentration of 100 mM).

### Lipid Preparation

DPPS (1,2-dipalmitoyl-*sn*-glycero-3-phosphoserine) and PI5P (D-myo-phosphatidylinositol 5-phosphate diC16) were purchased from Echelon Biosciences (Salt Lake City, UT, USA). DPPS was dissolved in DMSO and sonicated for one minute (using a sonicating water bath) and mixed by vortexing for 30 seconds, thereby forming a clear solution (333 µL DMSO per 1 mg DPPS). PI5P was suspended in DMSO and mixed by sonication and vortexing for several minutes (333 µL DMSO per 1 mg PI5P). A 2∶1 ratio of DPPS to PI5P was then made (500 µL of PI5P, 1000 µL of DPPS plus an additional 1500 µL of DMSO), and the resulting lipid mixture was sonicated and mixed by vortexing for several minutes. The resulting solution can be stored at -20°C and was found to be stable for at least six cycles of freeze/thaw.

On the day of the experiment, the lipid mix was thawed and mixed by sonication and vortexing for one minute. For each step up until the enzyme addition, each reagent addition was followed by a brief sonication and vortex mixing step. First, 1259 µL of the lipid mix was added to 315 µL of DMSO. Then, 5827 µL of buffer 1 (30 mM Hepes pH 7.4, 1 mM EGTA, 0.1% CHAPS) was added and lastly, 13525 µL of buffer 2 (46 mM Hepes pH 7.4, 0.1% CHAPS) was added. This lipid reagent was used for the assay as described below. All lipid mixtures were prepared in glass vessels to minimize surface absorption.

### Miniaturized Enzyme Activity Assay

PI5P4Kα (52.4 µL) was added to the lipid reagent (3 mL, described above) to form the enzyme-lipid mixture. First, 2 µL of the enzyme/lipid substrate reagent (2 µg/mL PI5P4Kα final concentration) were dispensed into a white solid-bottom 1536-well plate in columns 1–2, 4–48 using a Flying Reagent Dispenser (FRD, Beckman Coulter, Fullerton, CA, USA). Into columns 3 and 4, 2 µL of a no-lipid and no-enzyme control were dispensed, respectively, where the lipid was replaced by DMSO or the enzyme was replaced by buffer (20 mM Hepes 7.4, 0.1% CHAPS) in the enzyme-lipid mixture. Then, 23 nL of the library compounds and control compounds were transferred by a 1536 pintool (Kalypsys Systems, San Diego, CA, USA) into wells 5–48 and 1–4, respectively. To initiate the reaction, 1 µL of the ATP solution (20 mM Hepes pH 7.4, 60 mM MgCl_2_, 0.015 mM ATP and 0.1% CHAPS) was added. The final concentration of DMSO in the reaction was 5%. The resulting mixture was incubated at room temperature in the dark for one hour, at which time 2 µL of ADP-Glo reagent 1 were added to stop the reaction and remove any remaining ATP. After a 45-minute incubation, 4 µL of the ADP-Glo reagent 2 were added and allowed to incubate for 30 minutes. The luminescence was then read with a ViewLux high-throughput CCD imager (Perkin Elmer, Waltham, MA, USA). A total of six plates were assayed: one DMSO plate for monitoring the background trends and five concentrations of the LOPAC library (152, 76, 15, 3 and 0.6 µM final assay concentrations, respectively). The top concentration in the assay was attained by two sequential pin transfers of the top concentration of the compound library.

### Data Analysis

Screening data were corrected and normalized, and concentration-response curves were derived using in-house algorithms [25]. Overall assay performance, including trends in Z’ factor, % CV, and S/B, were recorded. Percent activity was computed after normalization using the median values of the uninhibited enzyme control (32 wells in column 1) and the no-enzyme, or 100% inhibited, control (32 wells, column 4). Additionally, the compound structures were evaluated, and the long chain compounds such as the C18 containing compounds from LOPAC^1280^ that are likely to act by disrupting the lipid vesicles were eliminated. Selected actives were procured for re-testing in the primary screening assay and for use in the counter assays and substrate competition assay.

### γ-^32^P-ATP Incorporation Assay and TLC

The PI5P4K assay was carried out as described in [10], [22], [23]. Briefly, the kinase reaction was carried out in a total of 50 µL of kinase buffer containing 20 µL of the resuspended lipids, 50 mM HEPES pH 7.4, 10 mM MgCl_2_, 10 µM non-radiolabeled ATP, 10 µCi [γ-^32^P]-ATP with 0.1 µg of GST-PI5P4Kα for 7 minutes at room temperature. The reaction was terminated by adding 20 µL of 4 N HCl. Phosphoinositides were extracted by adding 70 µL of methanol/chloroform (1∶1, vol:vol) mix and subjected to TLC (thin-layer chromatography) separation using heat-activated 2% oxaloacetate-coated silica gel 60 plates (20 cm×20 cm, EMD Chemicals Inc., Billerica, MA, USA) and a 1-propanol/2 M acetic acid (65∶35, vol:vol) solvent system. The radiolabeled product, PI(4,5)P_2_, was quantified with a Phosphorimager (Molecular Dynamics, STORM840, GE Healthcare, Waukesha, WI, USA).

### Counterscreen Assays

After hit confirmation of the selected and reacquired compounds in an 11-point retest in the qHTS ADP-Glo PI5P4Kα assay, the compounds were tested for their effect on the detection reagent. A firefly luciferase counterassay is available and has been used previously but the ADP-Glo kit has an additional enzyme that could be subject to compound interference, therefore a new counterassay was utilized [26]. The same assay design as above was used here except the lipid was replaced by DMSO and the enzyme was replaced by 20 mM Hepes pH 7.4, 0.1% CHAPS buffer. After 2 µL of the replica enzyme-lipid mix was added, compounds were transferred by pintool into this buffer. Then, an ADP/ATP mix representing 20% conversion levels (0.003 mM ADP and 0.012 mM ATP) was added. The two-step ADP-Glo detection kit was then used, and the luminescence was recorded as described above.

### Substrate Competition Assay

Seven concentrations of ATP (concentration in final reaction was 0.25 K_m_, 0.5 K_m_, 1 K_m_, 1.5 K_m_, 3 K_m_, 5 K_m_ and 10 K_m_, where K_m_  = 5 µM, the K_m_ of ATP) were used to assess the effect of ATP on the apparent IC_50_ of Tyrphostin I-OMe-AG-538. The experiment was otherwise conducted as described above for the miniaturized ADP-Glo enzyme assay.

## Results and Discussion

### Substrate Preparation

Preparation of the substrate is crucial for the development of a robust and miniaturizable lipid kinase assay. Phosphatidylserine (DPPS  = 1,2-dipalmitoyl-*sn*-glycero-3-phosphoserine) is often used as a carrier for lipid substrates and was used in combination with PI5P here (PI5P = D-myo-phosphatidylinositol 5-phosphate diC16) [17], [22], [27]. We aimed to make a DPPS/PI5P lipid mix in which the reagents would be stable for at least 8 hours and would not be subject to settling or clumping during the repeat dispensing steps. The main challenge originated from the diverging solubility and substrate competency trends by the lipids under consideration: PI5P and DPPS can have different alkyl chain lengths and the shorter ones (C4 and C8) are soluble in buffer but are not substrates for PI5P4Kα (data not shown and [17]), while the longer C16 PI5P4Kα substrates are not soluble in buffer but are soluble in DMSO.

Previously, a binding assay was developed with sucrose-loaded unilamellar vesicles that were then prepped by multiple freeze-thaw cycles followed by extrusion [18]. The study investigated a variety of lipid substrates and showed the importance of the negative charge for binding of the lipid to PI5P4Kα. Demian *et al*. previously established a 384-well enzymatic assay for PI5P4Kα and PI5P4Kβ using a translucent liposome-based presentation of the substrate (2∶1 ratio of phosphatidylserine to PI5P) [17]. They had used a liposome suspension in buffer for their C16 lipid substrate that was prepared by a time-consuming and technically challenging method that involved mixing the two lipids in acidified organic solvent, lyophilizing the mixture, and reconstituting it in buffer for 2-16 hours followed by repeated extrusion [17]. The authors utilized a commercial vendor to make the liposomes because the in-house preparations yielded variable results. That assay was used to screen a kinase-focused compound library, and a hit was identified and its binding to PI5P4Kβ was confirmed with additional biophysical characterization (IC_50_ = 0.58−1.4 µM depending on assay modality). During our attempts to adopt this protocol (without the use of an extruder to which we had no access) the substrate suspension was subject to rapid settling, and even after sonication and vortexing there were large visible particulates that were not amenable to 1536-well dispensing. Breaking liposomes down into smaller sizes has been shown to eliminate settling and perhaps an extruder step may have led to a particle size amenable to overnight stability and dispensing of small volumes without clogging the 1536-well dispenser [28]. The substrate characteristics of insolubility, settling, and the apparent necessity of commercial processing were not readily amenable to affordable and flexible substrate preparation and further assay miniaturization. Furthermore, large-scale robotic screening requires the creation of large and stable batches of reagents to enable continuous unattended robotic screening.

**Figure 2:**
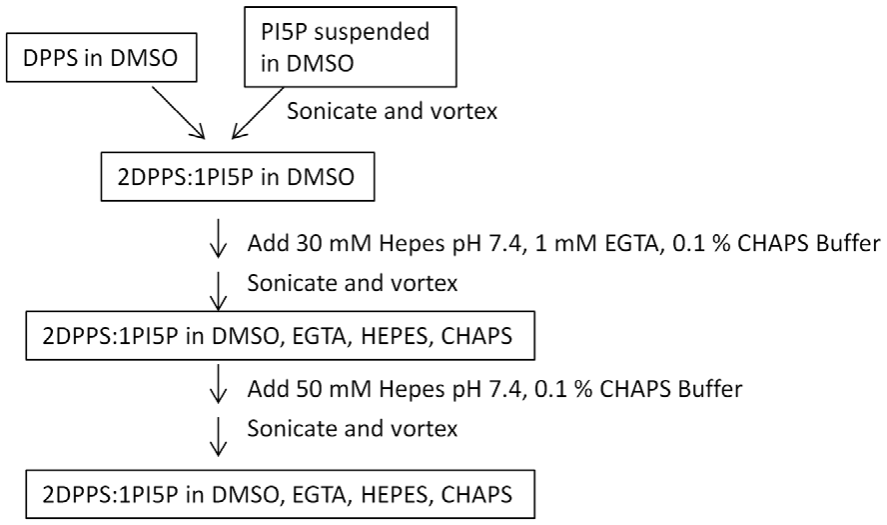
Schematic representation of the 2∶1 DPPS:PI5P lipid preparation protocol.

The method developed here allows the lipid substrate to be prepared at the bench without the need for expensive specialized equipment or processing. It was discovered that when the long-chain DPPS and PI5P lipid components were brought up in DMSO and mixed with just sonication and vortexing, a lipid-containing homogeneous solution was formed. This solution was not prone to settling and could be made reproducibly without the need for costly commercial processing. This PI5P/DPPS solution could then be added step-wise to buffer to yield a lipid solution that was not prone to settling and did not have any visible particulates. A flow chart describing the lipid substrate preparation is shown in Figure 2. The detergent CHAPS (0.1%) was used in the buffer to minimize enzyme, lipid, or compound sticking to plastic as well as to limit aggregation of the compounds to be tested [29]. The lipid substrate was found to be soluble in buffer with 5% DMSO. Further stability and scalability testing of this substrate preparation were performed during the assay and screen implementation as described in the following sections.

### Design and Miniaturization of the High-throughput PI5P4Kα Enzyme Assay

**Figure 3:**
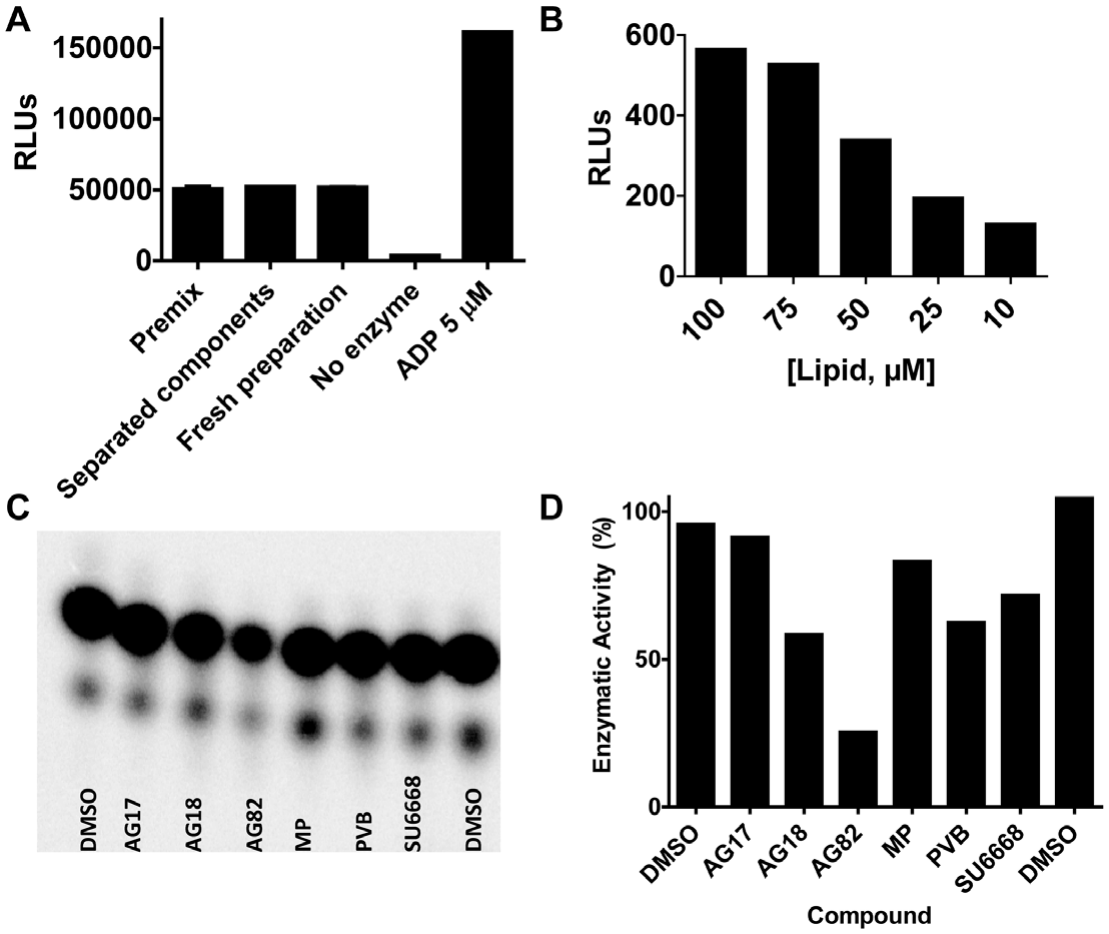
Lipid dependence, overnight stability and control compound. (A) The overnight (16 hour) stability of the assay reagents at 4°C when the enzyme and lipid were premixed, stored separately or made up fresh as compared to a no enzyme and 5 µM ADP (representing 0% and 100% conversion, respectively). The error bars represent the standard deviation (N = 2). (B) The PI5P lipid dependence of the PI5P4Kα enzyme reaction. The error bars represent the standard deviation (N = 2) and are not discernable on the plot. (C) and (D) Tyrphostin AG-82 (AG82) was identified as a weak inhibitor of PI5P4Kα (decreases the enzyme activity by 75%) by a radiometric assay that uses γ-^32^P-ATP and PI5P and measures the radiolabeled enzymatic product, PI(4,5)P_2_ after the separation by thin layer chromatography. Five additional compounds were tested and found not to significantly inhibit PI5P4Kα (AG17 =  tyrphostin AG-17, AG18 =  tyrphostin AG-18, MP = mycophenolate, PVB = purvalanol B and SU6668). All compounds were tested at 100 µM, except for PVB, which was tested at 10 µM due to solubility limitations at higher concentrations. The raw image and the extracted data are shown in (C) and (D), respectively. The commercial PI5P substrate predominantly contains two palmitate groups with a very small amount of deacylated lipid lyso-PI5P that contains only one palmitate group. The intense top spots in (C) represent the PI(4,5)P_2_ product with two palmitate groups, and the faint spots below represent the product with just one palmitate group.

To enable large-scale library screening, a 1536-well luciferase-coupled bioluminescence PI5P4Kα assay was pursued. Our goal was to design an enzymatic reaction whereby the enzyme, lipid substrate, and ATP could be dispensed in two, rather than three, distinct steps to minimize the overall variability of dispense, which in turn required the combination of two of the reagents into one vessel. Many kinases, including PI5P4Kα have low levels of substrate-independent consumption of ATP, which precluded the enzyme and ATP from being stored together. There was also a spontaneous phospho transfer from ATP (by a very small amount) to the lipid when these two reagents are stored together (data not shown). The stability of the enzyme/lipid solution was tested, and it was found that there was a less than 5% change in activity for a premixed solution maintained at 4°C for 16 hours versus a freshly prepared stock (see Figure 3A). Stability over 8 hours is ideal for the large robotic screens to allow for facile reagent exchanges to be scheduled in a continuous robotic run. This reagent configuration (enzyme/lipid followed by ATP) was used in the assay.

**Figure 4:**
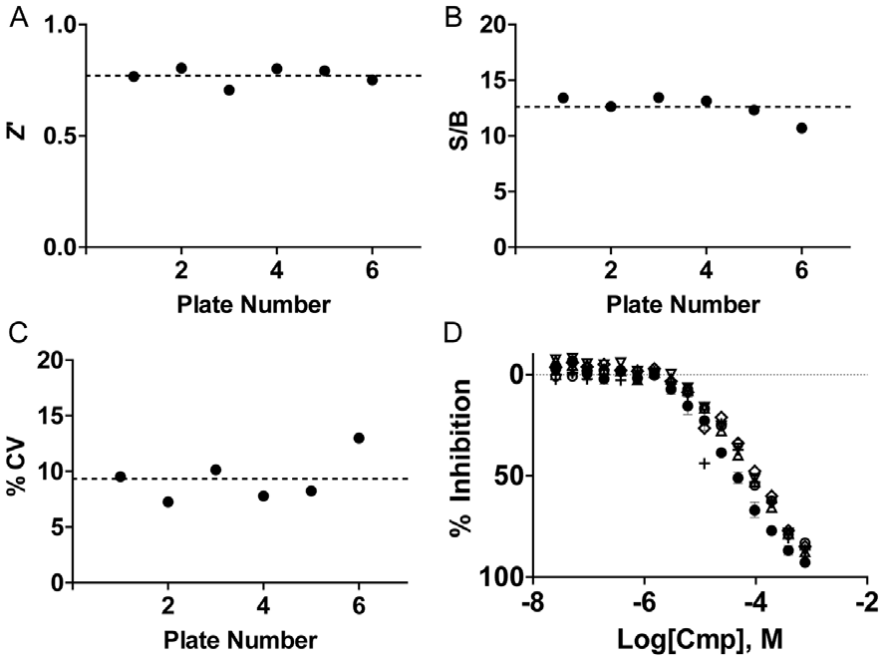
Performance of the PI5P4Kα assay in 1536-well assay plates. (A) Z’ factor, (B) signal/background, and (C) % column variance as a function of assay plate. An average Z’  = 0.77±0.04, S/B  = 12.6±1.04 and % CV  = 9.32±2.1 was achieved for the six plates (reported as average ± standard deviation). (D) IC_50_ data for the control compound AG-82 on the six assay plates depicted with six symbols. MSR of the IC_50_ = 1.29. Each plate contained 2 16-pt titrations, which were averaged, and the standard deviation is depicted as error bars on the plot (N = 2).

The K_m_ of ATP (5–6 µM) had been determined previously [17], [30], and the ATP concentration was set at the K_m_ to ensure that the assay would be sensitive to ATP-competitive inhibitors. The substrate concentration was determined such that a reasonable % ATP conversion (<20%) and robust assay statistics could be obtained with a one-hour incubation time by 10 nM of PI5P4Kα (see Figure 3B and Figure 4). A PI5P concentration of 75 µM was determined to be optimal because 50 µM resulted in lower enzyme activity and 100 µM gave nearly the same results as 75 µM. The K_m_ of this substrate is unknown and could not be measured here because the use of lipid concentrations much higher than 100 µM, required for the accurate derivation of K_m_, would lead to a DMSO concentration that is not well tolerated by the enzyme (see following section). By having excess lipid relative to the S_0.5_ (∼50 µM), a slight loss of lipid sticking to the storage bottle, dispenser tubing or assay plate during the HTS can be tolerated without an appreciable change in assay performance. The two-step ADP-Glo kit was used to detect the ADP product, which allows for the development of a sensitive assay without the need for high levels of conversion [31]. The detailed assay protocol can be found in the Materials and Methods and is summarized in Table 1. The signal to background of the PI5P4Kα assay was quite comparable when the substrate was prepared by the method of Demian *et al*. and the method described here (S/B  = 12 -13, Figure S1) [17], indicating that the new lipid preparation could function as a substrate for PI5P4Kα. Additionally, the lipid mixture was found to be stable to at least six rounds of freeze/thaw (data not shown), which allows the substrate to be prepared and validated in large batches and used for multiple experiments.

**Table 1:** Steps of the High-Throughput PI5P4Kα Assay.

Step	Parameter	Value	Description
**1**	Enzyme/Lipid	2 µL	Enzyme/lipid, no enzyme and no lipid solutions; reagent bottles are kept on ice
**2**	Centrifugation	10 sec	Spin 300×g
**3**	Library and Control Compounds	23 nL	152, 76, 15, 3 and 0.61 µM final concentration titration series of library and 769 µM AG-82 1:2 16-point control titration.
**4**	Substrate	1 µL	ATP, room temperature
**5**	Incubation time	60 min	Room temperature
**6**	ADP-Glo Reagent 1	2 µL	ADP-Glo detection reagent 1 at room temperature
**7**	Incubation time	40 min	Room temperature
**8**	ADP-Glo Reagent 2	4 µL	ADP-Glo detection reagent 2 at room temperature; reagent bottle is protected from light
**9**	Incubation time	30 min	Room temperature
**10**	Assay Readout	Luminescence	ViewLux in end-point mode: 20 second exposure

Step Notes.

1. Dispensed into white solid-bottom 1536-well MB Greiner plates with a Flying Reagent Dispenser (FRD). Reagent is kept on ice. Final concentration is 10 nM PI5P4Kα, 75 µM PI5P and 150 µM DPPS.

2. Plates centrifuged at 300×g for 10 seconds.

3. DMSO compound solutions transferred with a Kalypsys pintool.

4. Dispensed with a FRD. Final concentration is 5 µM ATP.

5. Plates incubated at room temperature.

6. Dispensed with a FRD.

7. Plates incubated at room temperature.

8. Dispensed with a FRD.

9. Plates incubated at room temperature.

10. Luminescence was measured with a ViewLux CCD Imager with a 20 second exposure time.

### DMSO Tolerance

The effect of DMSO on the enzyme reaction was tested, and it was found that up to 7.25% DMSO there was little effect on the enzyme activity and assay performance. At high DMSO levels, the concentration of DMSO used to make the enzyme-lipid reagent was found to impact the ratio of lipid-coupled kinase ATPase activity to lipid-independent kinase ATPase activity: 5% DMSO was found to have very little lipid-independent kinase ATPase activity (∼4%) while allowing the lipid substrate to be readily accessible to the enzyme active site; however, at DMSO concentrations of greater than 15%, the amount of lipid-independent phosphorylation increased dramatically (See Figure S2). Therefore, DMSO at 5% was able to help solubilize the substrate while retaining the desired substrate-coupled enzyme activity, and this concentration of DMSO was used for the miniaturized luciferase-coupled assay.

### Pilot Screen

Validation of the PI5P4Kα assay was performed by a qHTS (quantitative HTS, [25]) against the library of pharmacologically active compounds (LOPAC^1280^, Sigma-Aldrich) arrayed as a five-point titration series (152, 76, 15, 3, and 0.61 µM final compound concentration). Additionally, a vehicle only DMSO plate was run and used to monitor the background. The validation experiment showed excellent performance as measured by a stable signal:background ratio (12.6), stable Z’ factor (0.77) and a low CV (9.3%) (Figure 4A–C). Tyrphostin AG-82 had been identified by a radiometric assay that uses γ-^32^P-ATP and PI5P and measures the radiolabeled enzymatic product, PI(4,5)P_2_, after separation by thin layer chromatography. AG-82 displayed ∼75% inhibition of activity at 100 µM in the radiometric assay (See Figure 3C) and was used as a control compound here. It exhibited an excellent MSR (Minimum Significant Ratio, [32]) of 1.29 (Figure 4D) in the LOPAC^1280^ screen and yielded an IC_50_ of 93 µM, which is consistent with the observed 75% inhibition at 100 µM in the radioassay. Importantly, the enzyme-substrate solution was not prone to settling and did not require mixing during dispense of multiple plates, as evidenced by the successful scale-up experiment performed on a fully-automated robotic platform (See Figure S3). Curve fitting of the concentration responses using the Hill equation was performed using in-house methods and is detailed in the Materials and Methods section. Of the 1,280 LOPAC compounds screened, a total of 32 compounds were identified with upper and lower asymptotes in their concentration responses and an efficacy of over 80%.

**Figure 5:**
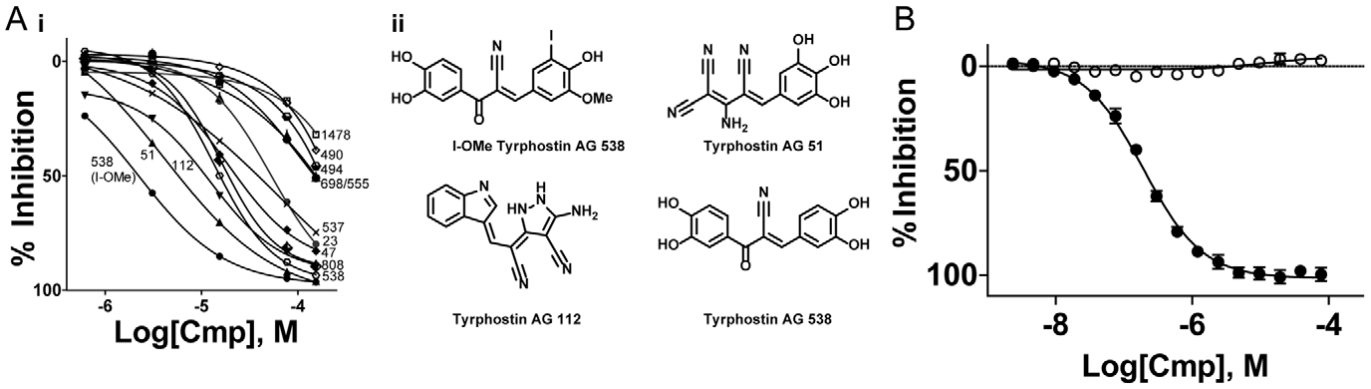
Confirmation of inhibitors. (A) *i*, The IC_50_ inhibition curves of the tyrphostin analogues identified from the Lopac library are shown. Numbers refer to the tyrphostin analog and IC_50_s were determined to be as follows: tyrphostin I-OMe-AG-538 (2 µM), tyrphostin 51 (5 µM), tyrphostin AG 112 (13 µM), tyrphostin AG 538 (14 µM), tyrphostin AG 808 (18 µM), tyrphostin 47 (20 µM), tyrphostin AG 537 (32 µM), tyrphostin 23 (45 µM), tyrphostin AG 555 (50 µM), tyrphostin AG 698 (50 µM), tyrphostin AG 490 (89 µM), tyrphostin AG 494 (89 µM), tyrphostin AG 1478 (100 µM). *ii*, Structures of the four most potent tyrphostin analogs. (B) The IC_50_ curves with standard deviation error bars (N = 2) of tyrphostin I-OMe-AG-538 in the PI5P4Kα assay (squares) and the counterscreen (open circles).

The LOPAC^1280^ library contains many tyrphostins and indeed several analogues of the AG-82 (Tyrphostin 25) control with a range of potencies (IC_50_s of 2.5 µM to over 100 µM, Figure 5A) were identified. The most potent analogue, I-OMe-tyrphostin AG-538 (I-OMe-AG-538), had an IC_50_ of 2.5 µM. Additionally, long-chain compounds, such as 1-O-Octadecyl-2-O-methyl-sn-glycero-3-phosphorylcholine, were identified as inhibitors. These compounds may be acting through prevention of lipid substrate binding to PI5P4K.

Seven of the initial hits were reacquired and tested as 11-point titrations in the PI5P4Kα enzyme assay. The activity of all of these inhibitors was reconfirmed indicating the reproducibility of the assay. The IC_50_ was determined to be 1 µM for a freshly plated stock of I-OMe-AG-538.

### Counterscreen Assay on Selected Hits

When developing a screen, it is important to assess the type of compounds that could interfere with the detection. For the assay developed here, the formation of the product ADP is detected by a coupled assay system that contains several enzymes [31], [33], [34]. Compounds that impact these detection components could be erroneously identified as actives, so a facile assay was developed to test for compounds that interfere with this detection system. Previously, compounds have been identified that interact with the firefly luciferase system, one of the components in the ADP-Glo kit [35]. It is possible and indeed recommended to screen apparent hits in a luciferase-coupled assay against the luciferase enzyme itself [36]. Furthermore, ADP-Glo contains additional enzymes that may also be subject to compound interference, making a counterscreen for any apparent hits using the full ADP-Glo kit very important. The selected compounds from the screen were tested in an assay where a fixed concentration of ADP/ATP, representing 20% conversion, is present in the absence of the enzyme and lipid, and the detection system was used to quantitate the ADP. If the compound does not act on the detection system, the amount of luminescence, which correlates with the amount of ADP, should not differ between wells with compound and wells with DMSO. If, however, the compound interferes with the detection system, apparent IC_50_ values would be obtained for the compound titration. The compounds were found to be free of effects against the detection in the ADP-Glo detection system (Figure 5B), indicating that the compound IC_50_ values that were obtained in the PI5P4Kα assay were due to the effect of the compound on the enzyme reaction itself and not on the detection reagents.

### ATP Competition

**Figure 6:**
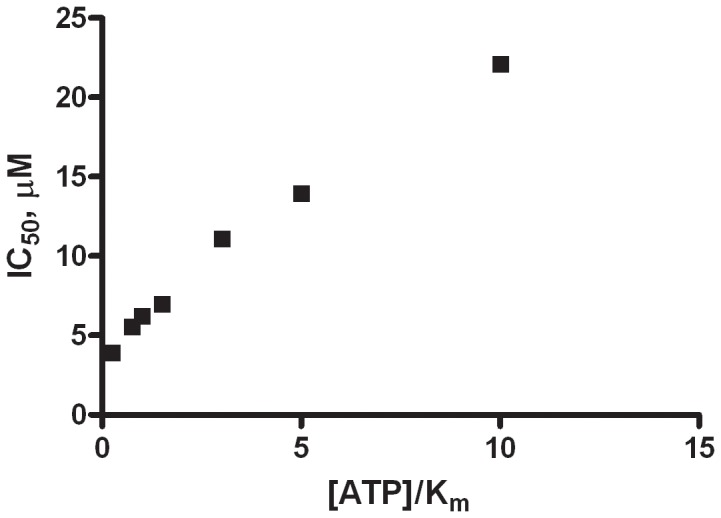
ATP competition with tyrphostin I-OMe-AG-538. The IC_50_ of tyrphostin I-OMe-AG-538 for PI5P4Kα is plotted against the [ATP]/K_m_. The K_m_ of ATP is 5 µM, and seven concentrations were evaluated.

To help identify the mode of inhibition of I-OMe-AG-538, the IC_50_ was measured using the miniaturized ADP-Glo assay in the presence of seven concentrations of ATP spanning 0.25xK_m_ to 10×K_m_ of ATP. By the slope of the resulting data plotted as IC_50_ vs. [ATP]/K_m_, the inhibitor can be determined to be a non-, un- or competitive inhibitor with ATP [37]. Figure 6 shows a positive relationship between the [ATP]/K_m_ and the IC_50_ indicating competitive inhibition, i.e. that the inhibitor and ATP cannot bind to the enzyme in an orthosteric mode. Additionally, the K_i_ was determined to be 0.5 µM. Tyrphostin AG-538 (AG-538) inhibits insulin-like growth factor 1 receptor (IGF1R) with an IC_50_ of 60 nM and is competitive with respect to substrate for IGF1R [38]. Both AG-538 and I-OMe-AG-538 inhibited IGF1R in a similar dose-dependent manner in a cell assay [38]. Modeling showed that the tyrosines in the substrate overlaid with the catechols in AG-538 and that AG-538 mimics the substrate [38]. For PI5P4Kα, however, I-OMe-AG-538 does not resemble the substrate PI5P. Indeed, tyrphostins have been shown previously to be ATP competitive, substrate competitive and competitive with both substrate and ATP [39], [40]. Although we did identify a few compounds such as oleamide and OMDM-2 ((R)-N-oleoyl Tyrosinol), which were found to be weak inhibitors (IC_50_s ∼30 µM), whose potency did not vary with ATP, and could therefore interact with the lipid pocket, we were not able to vary lipid in the present assay configuration, due to the limited solubility of the lipid substrate. As well, if the lipid concentration was increased to look for competition, the DMSO concentration would increase, and the enzyme would uncouple from its substrate, making it impossible to conduct this experiment. To assess lipid competition, either a higher concentration of substrate in DMSO would need to be tried or the commercial liposome preparation process could be utilized. Alternatively, methods, such as surface plasmon resonance using lipid-coupled sensor chips, could be employed to determine the mechanism of action in regards to substrate competition.

### Conclusions

In conclusion, this new and validated luciferase-coupled bioluminescence 1536-well PI5P4Kα assay entails a lipid preparation that is more facile and less costly than earlier assay designs for the important drug targets PI5P4Kα/PI5P4Kβ. The lipid could be solubilized in DMSO with just sonication and vortex mixing on the benchtop. The enzyme/lipid mixture is stable overnight and did not require additional stirring during the course of our screens. The IC_50_ of the control compound measured in this assay was corroborated by the orthogonal thin layer chromatography radiolabeled ATP assay. Inhibitors were identified in the miniaturized ADP-Glo assay that reconfirmed upon reacquisition, and they were found to be free of interference with the detection system. The tyrphostin molecule I-OMe-AG-538 was shown to be competitive with respect to ATP for binding to PI5P4Kα and to have an IC_50_ of 1 µM. This new facile substrate preparation and assay method can be expanded to other lipid kinase family members for which the more labor-intensive liposome methods have been used and should enable the large-scale screening of libraries, such as the Molecular Libraries Small Molecule Repository (MLSMR) library that contains nearly 400,000 compounds.

## Supporting Information

Figure S1 **Assay performance comparison.** Comparison of assay performance of the luciferase-coupled assay system using the lipid prepared in DMSO and the lipid prepared from lipid cakes. The luminescence from enzyme and no enzyme preparations using the lyophilized lipid cakes sonicated in buffer method (black) and the DMSO method (checkered) are shown. Standard deviation error bars are shown (N = 2).
(TIF)

Figure S2 **DMSO effect on the performance of the luciferase-coupled assay system.** DMSO concentrations were tested from 5–30%. Testing below 5% DMSO was not feasible due to the requirement of DMSO for solubilization of the substrate. Standard deviation error bars are shown (N = 2).
(TIF)

Figure S3 **Assay performance on robotic system.** Stable performance of the PI5P4Kα assay was obtained in the 1536-well assay scale-up experiment performed on a fully-automated robotic platform [41] (147 plates tested). The Z’ factor is shown as a function of assay plate.
(TIF)
