# Supplementary figures and images for: A Homogeneous, High-Throughput Assay for Phosphatidylinositol 5-Phosphate 4-Kinase with a Novel, Rapid Substrate Preparation
Source: PLoS One. 2013 Jan 10;8(1):e54127. doi: 10.1371/journal.pone.0054127 (PMC3542272; doi:10.1371/journal.pone.0054127)

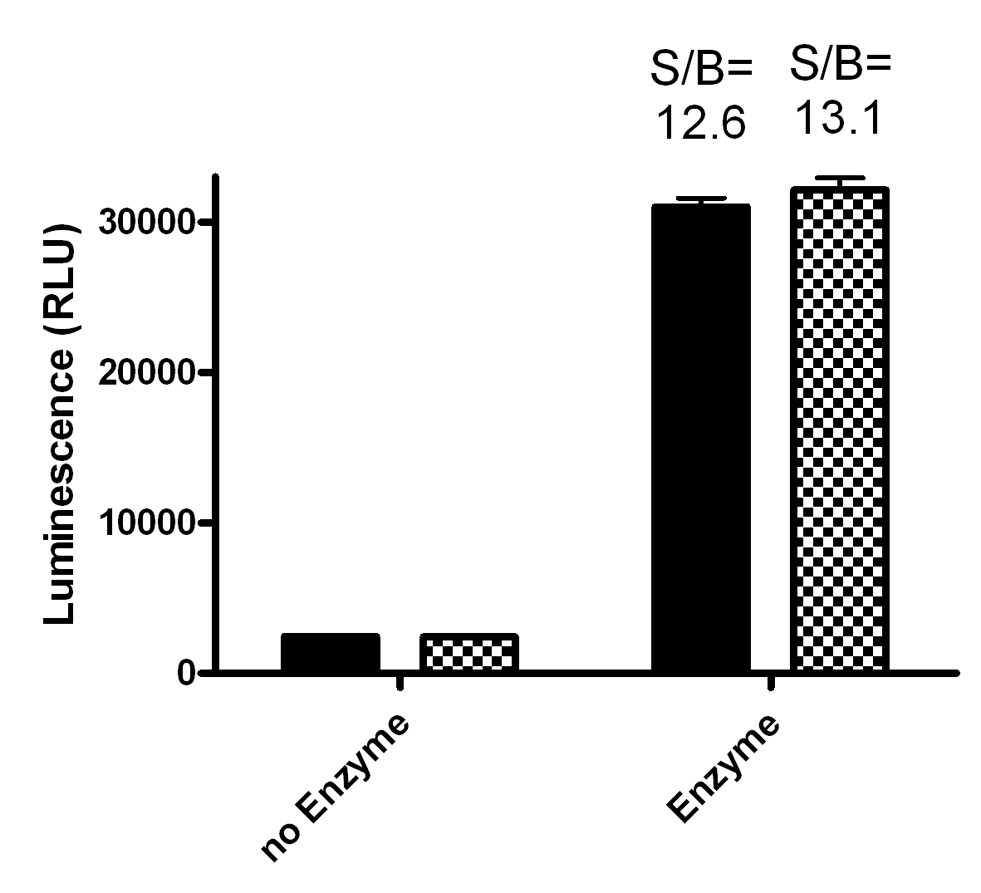

Supplement: Figure S1 — Assay performance comparison. Comparison of assay performance of the luciferase-coupled assay system using the lipid prepared in DMSO and the lipid prepared from lipid cakes. The luminescence from enzyme and no enzyme preparations using the lyophilized lipid cakes sonicated in buffer method (black) and the DMSO method (checkered) are shown. Standard deviation error bars are shown (N = 2). (TIF) [file pone.0054127.s001.tif]

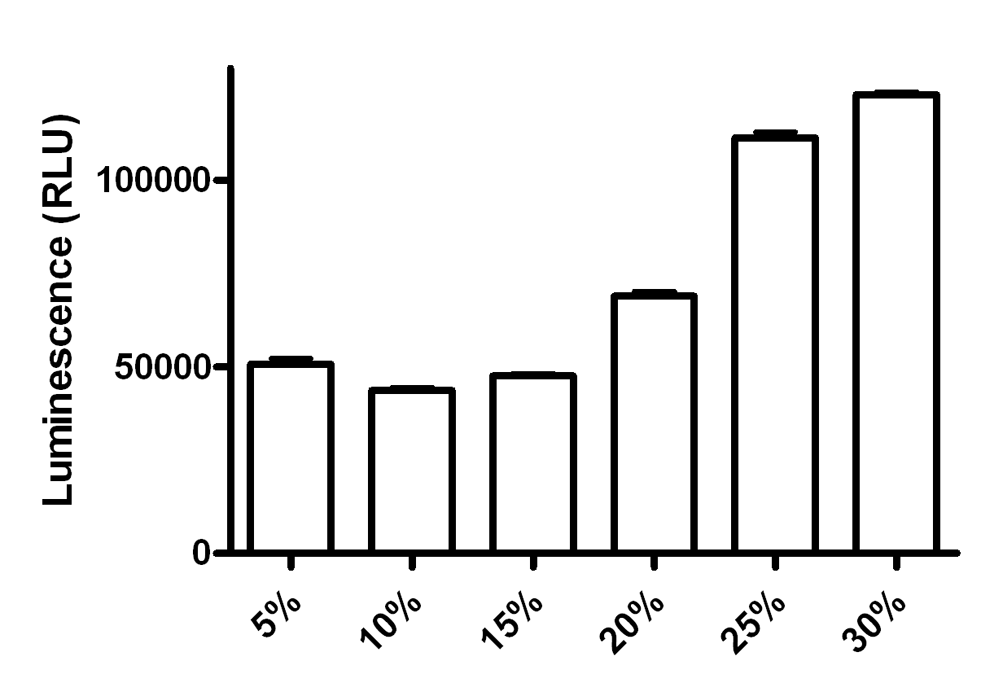

Supplement: Figure S2 — DMSO effect on the performance of the luciferase-coupled assay system. DMSO concentrations were tested from 5–30%. Testing below 5% DMSO was not feasible due to the requirement of DMSO for solubilization of the substrate. Standard deviation error bars are shown (N = 2). (TIF) [file pone.0054127.s002.tif]

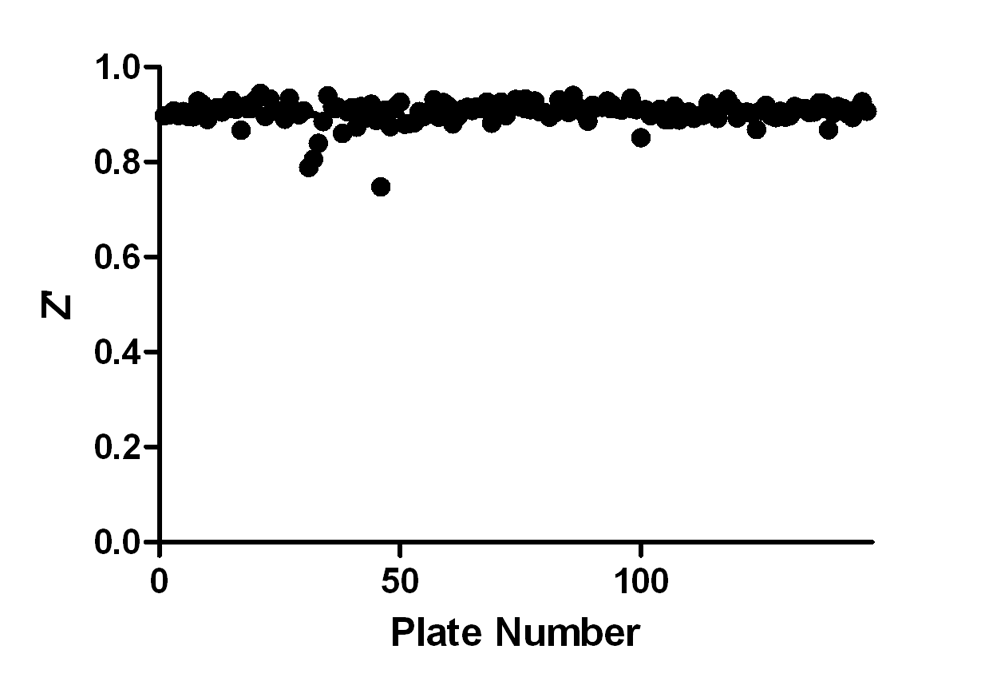

Supplement: Figure S3 — Assay performance on robotic system. Stable performance of the PI5P4Kα assay was obtained in the 1536-well assay scale-up experiment performed on a fully-automated robotic platform [41] (147 plates tested). The Z’ factor is shown as a function of assay plate. (TIF) [file pone.0054127.s003.tif]
